# Enhancing multi-sectoral collaborations for the prevention and control of NCDs in Thailand with a new approach

**DOI:** 10.1186/s12961-024-01262-z

**Published:** 2024-12-18

**Authors:** Bundit Sornpaisarn, Somsak Chunharas, Sarnti Sornpaisarn, Pairoj Saonuam, Rifat Farzan Nipun, Chaniphun Butryee, Bhubate Samutachak, Maneekwan Chandarasorn, Nattapon Supadulya, Suttikarn Chunsuttiwat, Sumonmarn Singha, Wiwat Rojanapithayakorn, Kumnuan Ungchusak, Jürgen Rehm

**Affiliations:** 1 Institute for Mental Health Policy Research, Centre for Addiction and Mental Health, Room 916, 250 College Street, Toronto, ON M5T 1R8 Canada; 2 Dalla Lana School of Public Health, University of Toronto, 155 College Street, Toronto, ON M5T 1P8 Canada; 3 National Health Foundation, Thailand, 1168 Soi Phaholyothin 22, Phaholyothin Road, Chatuchak, Bangkok, 10900 Thailand; 4 Faculty of Medicine, Dentistry and Health Sciences, University of Melbourne, 161 Barry Street, Carlton, VIC 3010 Australia; 5 Thai Health Promotion Foundation, 99/8 Soi Ngamduphli, Sathon, Bangkok, 10120 Thailand; 6 Institute of Nutrition, Mahidol University, 999 Phutthamonthon 4 Road, Putthamonthon, Nakhon Pathom, 73170 Thailand; 7 Institute for Population and Social Research, Mahidol University, 999 Phutthamonthon 4 Road, Putthamonthon, Nakhon Pathom, 73170 Thailand; 8 Fiscal Policy Office, Ministry of Finance, Phraram 6 Road, Phaya Thai, Bangkok, 10400 Thailand; 9 Health Technical Office, Ministry of Public Health, Nonthaburi, Thailand; 10 Department of Diseases Control, Ministry of Public Health, Nonthaburi, Thailand

**Keywords:** Knowledge broker, Knowledge generation and dissemination, Knowledge translation, Multi-sectoral collaboration (MSC), Non-communicable diseases (NCDs), Stakeholder involvement, Sustainable Development Goals (SDGs)

## Abstract

**Background:**

To achieve the Sustainable Development Goals (SDGs) by 2030, Thailand must engage in effective multi-sectoral collaboration (MSC). However, implementing MSC in Thailand presents significant challenges. Although Thailand had a 2011–2020 MSC strategic plan for the control of non-communicable diseases (NCDs) with the prime minister taking the lead, joined by many non-health ministers, not a single meeting was called over those 10 years. This paper describes the development of a new tool created to enhance MSC between health and non-health sectors in controlling NCDs in Thailand. Stakeholder-engaged research will be used to implement and evaluate this tool. This paper also describes the research planned to test the new approach.

**Methods:**

The authors used two main methods: (1) a narrative review on MSC enhancement and (2) a series of four consultation meetings with key stakeholders – in the health, non-health and academic sectors – to develop a research study to implement and evaluate the new approach.

**Results:**

To address previous MSC implementation problems, the proposed novel MSC enhancement approach emphasizes three principles: (1) pursuit of committed-stakeholder involvement at the middle-management level, instead of relying on the top-management level, an approach which has never been successful; (2) production of knowledge to support specific, achievable target policies; and (3) use of a comprehensive set of knowledge-translation activities and knowledge brokers to solve the problem of ineffective routine official communications between members of the MSC. Using participatory consultations during the research proposal development, middle-level officials from three non-health ministries (the Ministries of Agriculture, Finance and Education) agreed to join the MSC to work together to solve specific problems regarding the control of NCDs. A target-advocated policy for each ministry was formulated and agreed upon by both non-health-sector and health-sector stakeholders.

**Conclusions:**

This new approach (middle-management oriented), if implemented, may encourage more commitment from the Ministries’ representatives, policy-relevant knowledge generation and effective communications between ministries involved in an MSC. Ideally, it would complement the conventional approach (top-management oriented) in enhancing the MSC for controlling NCDs, and thereby bring hope for achieving the NCD-related SDGs for Thailand and possibly other countries as well.

## Introduction

Non-communicable diseases (NCDs) are the leading cause of premature death globally. To respond to this, numerous world leaders who attended the 2015 United Nations High-level Political Forum pledged to reduce premature deaths from NCDs by a third by 2030 [1]. Due to the complexity of the interplay between the various determinants of health, multi-sectoral collaboration (MSC) which extends beyond the healthcare sector is crucial to the establishment and implementation of NCD prevention and control policies [2, 3].

A simple but clear definition of a successful MSC is a “synergistic alliance” between involved stakeholders [4], but factors facilitating successful or unsuccessful MSCs are more complicated. Factors enabling successful MSC include a supportive environment (e.g. political commitment), a clear shared purpose across sectors (win–win strategies), relevant stakeholder engagement, positive coordination and communication, information production and sharing, ensuring resources, and evaluation and learning [2–5]. For example, unlike the conventional methods currently used for communication between collaborating ministries, a set of comprehensive communication strategies (e.g. using communications both through general means and face-to-face meetings as well as addressing knowledge management from both knowledge producer and knowledge user sides) as proposed by Levis et al. in 2006 would increase the likelihood of effective communication and resulting in improved MSC performance [6].

As with many initiatives, however, MSC comes with its own set of challenges. On the basis of their umbrella review, Amri et al. summarized five barriers that made for an unsuccessful MSC: a lack of a shared vision across sectors, a lack of funding, a lack of political leadership, a lack of ownership and accountability, and insufficient and unavailable indicators and data [2]. Moreover, the development of a multi-sectoral action plan is a crucial first step, and any challenges beyond its development would only arise once countries began to implement it [5, 7]. An example of such a failure during implementation would be if sectors sent representatives with no actual decision-making power to MSC meetings [5].

NCDs and their risk factors generate significant burden to Thailand. In 2014, 368,872 Thai people died from NCDs, which accounted for 67% of total deaths [8]. The causes of death included cardiovascular diseases (122,581 deaths), cancers (96,988 deaths), diabetes mellitus (30,529 deaths) and chronic respiratory diseases (22,531 deaths) [8]. Prominent NCD risk factors in Thailand include tobacco smoking, which was attributable in 54,610 deaths; alcohol drinking, attributable in 21,843 deaths; unhealthy diets, attributable in 21,650 deaths; and physical inactivity, attributable in 11,453 deaths [9]. The total number of disability-adjusted life years (DALYs) lost due to NCDs in Thailand in 2014 was 10.5 million years, accounting for 70% of the total DALYs lost from all diseases combined [10].

Evidence showed that the Thai government fully adopted the MSC approach; however, a major complex obstacle – implementation failure – has hindered the Thai government’s attempt to execute multi-sectoral action plans to combat NCDs as recommended by the WHO [11, 12]. There were three main events supporting this argument. First, the Thai government endorsed the Thailand Healthy Lifestyle Strategic Plan, 2011–2020, which aimed to promote healthy lifestyles to prevent NCDs among Thais [13]. This strategic plan involved the creation of a comprehensive national multi-sectoral committee, with over 10 non-health ministers serving as members and the prime minister serving as the chairperson. Unfortunately, a committee meeting was never called [14], reflecting the lack of real political commitment to implement the MSC, similar to what has happened in many other countries [7]. Second, WHO-Thailand and the Royal Thai Government collaborated on creating a formal agreement called the WHO Country Cooperation Strategy that began in 2002 and covered several health issues and began including a strategy for the reduction of NCDs (CCS-NCDs) in 2012. The CCS-NCDs strategy for 2012–2016, which aimed to strengthen MSC for NCD control in Thailand, was deemed ineffective by an official evaluation process because it was more technical in nature than policy-oriented [14]. The second iteration of the CCS-NCDs strategy moved towards including more policy work but was still largely confined to public health activities. Third, the United Nations Inter-Agency Task Force on the Prevention and Control of Noncommunicable Diseases visited Thailand to evaluate and learn from the Thai experience in enhancing health promotion and preventing and controlling NCDs in August of 2018 [15]. The mission reported that Thailand had done well on many fronts, but one crucial recommendation this task force made was to enhance MSC by appointing the prime minister as the chairperson of an official MSC committee [15]. However, Thailand had already attempted such an implementation using a similar structure (top-management approach), and it had ultimately been unsuccessful. The United Nations Thematic Working Group on NCD Prevention and Control (UNTG) was then established to complement the work of the Thai Government by facilitating multi-sectoral action for an implementation of the recommendations of the UNIATF mission. The report of the results of its 2 years of work results mentioned a number of major challenges; two of them were (1) the UNTG had no formal authority to enforce follow-up activities and thus had to rely on voluntary commitment of stakeholders and (2) the high-level officials from the non-health ministries reduced their participation over time [16].

All these pieces of evidence reflected that although MSC may sound good in theory, it does not necessarily guarantee a successful implementation, especially without high-level political commitment (see [5, 16]).

Thus, an effective strategy should be developed that ensures that key stakeholders, regardless of high-level political commitment, are keen to participate in the NCD prevention and control effort. Hence, the Centre for Addiction and Mental Health in Canada, as well as the National Health Foundation and the Thai Health Promotion Foundation in Thailand, formed a planning team (made up of the first five and final authors of this article) to conduct a literature review and consult with key stakeholders in Thailand to design a novel MSC enhancement approach and a research proposal to implement and test it. Funding for this planning project was granted by the Canadian Institute of Health Research (CIHR) in 2020 for the purpose of developing a research proposal that was relevant to a real-world context, feasible to implement and had solid commitments by all key stakeholders. This article aims to describe the processes and outputs of these efforts.

## Methods

**Fig. 1:**
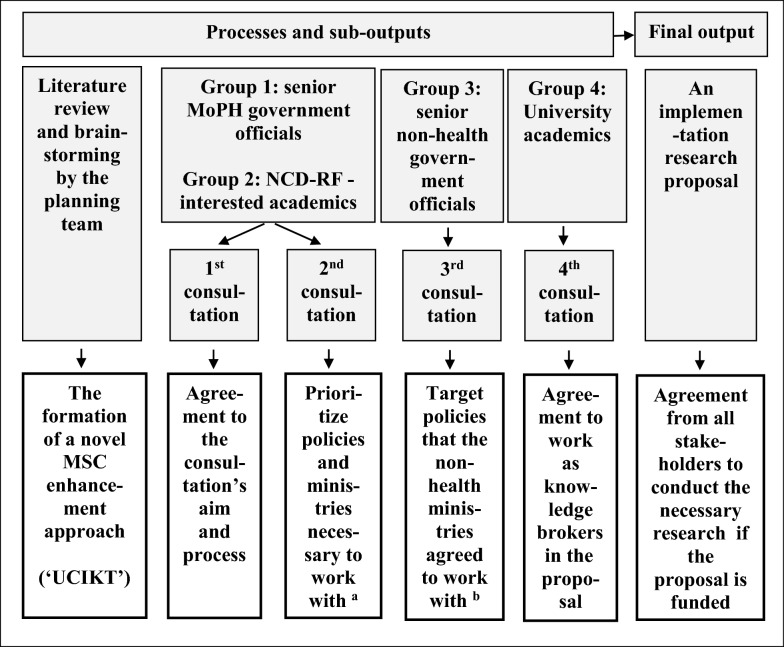
Conceptual framework of the processes and outputs in developing the concept of and a research proposal to implement and test the novel MSC enhancement approach. MoPH, Ministry of Public Health; MSC, multi-sectoral collaboration; NCD-RFs, non-communicable diseases risk factors; UCIKT, user-oriented comprehensive integrated knowledge translation. ^a^Details are in Table 1. ^b^Details are in Table 2

The planning team conducted a narrative review and a series of four consultation meetings with key stakeholders during 2020–2021. See Fig. 1 and detailed explanations below for further details.

### Narrative review

The narrative review addressed the following questions: Does MSC work effectively in practice? What are the challenges associated with it? What are the best practices for developing a successful MSC? How does knowledge translation (KT) play a role in evidence-based policy advocacy and in enhancing MSC? And, lastly, what are the best practices for KT in improving MSC? We searched two online databases (PubMed and Google Scholar) using search terms such as non-communicable diseases and multi-sectoral collaboration, multi-sectoral action, multi-sectoral collaboration enhancement, success and failure of multi-sectoral collaboration, knowledge translation and stakeholder involvement to retrieve published articles and policy documents written in English with no date restrictions. We also searched gray literature related to our topics written in the Thai language. After the reviews were completed, the planning team brainstormed and discussed formulating a novel MSC enhancement approach. We applied the MSC theory of “stages in the process of developing a multi-sectoral action plan to address non-communicable diseases” proposed by ref. [5], and the concepts of knowledge translation [17] and knowledge brokers [18] to guide the development of our approach. We then developed a stakeholder-engaged proposal to implement and test it.

### A series of consultation meetings

#### Stakeholders involved in the consultation process

Our consultation process engaged four distinct groups of individuals (see Fig. 1). These included: (1) senior government officials in the Ministry of Public Health (MoPH) working on the prevention of NCDs and/or relevant risk factors, individuals in this group were people who typically attempt to influence non-health ministries to implement policy measures in the prevention and control of NCDs; (2) academics with research interests in alcohol, tobacco, unhealthy diet and physical inactivity, the work of this group of individuals typically supports the MoPH in their MSC efforts to advocate for specific health issues; (3) middle-level government officials outside of the MoPH who typically provide input for the policy decision-making processes in their respective ministries; and (4) academics who were health-oriented yet capable of directing their efforts towards a non-health ministry’s agenda. For example, rather than being exclusively focussed on specific health initiatives such as the creation of tobacco- or alcohol-free schools, these selected academics were able to support a broader concept of school health and wellness for the Ministry of Education.

#### A consultation process

We conducted four stakeholder consultation meetings in Thailand to plan the stakeholder-engaged research proposal (see Fig. 1). The first consultation meeting introduced the goal of the proposal-planning process to health-sector stakeholders, including groups 1 and 2 mentioned above. It aimed to get the stakeholders’ commitments to the project and to brainstorm ideas for implementing and testing the novel MSC enhancement approach. Groups 1 and 2 also participated in the second meeting, which aimed to identify priority NCD risk factors and their respective prevention and control policies that required cooperation from non-health ministries.

The third set of consultation meetings involved a one-on-one meeting between the planning team and the senior government officials from each of the three target Ministries: the Ministry of Agriculture (MoA), the Ministry of Finance (MoF) and the Ministry of Education (MoE). These people were in group 3. They were chosen for their perceived interest in NCD prevention and control. Each non-health government official had the authority to select specific health policy topics they wished to engage in with from the priority policy list resulting from the second meeting. In the fourth and final set of meetings, we invited three university academics (group 4) to agree to work as knowledge brokers for the UCIKT in the future and consulted with each of them separately with the goal of developing the final draft of the research proposal.

#### Ethical issues/statement

This article discussed the results of a research proposal development process. As it was not a research study, it did not require ethics approval.

## Results

### The novel MSC enhancement approach

The theory of “stages in the process of developing a multi-sectoral action plan to address non-communicable diseases” proposed seven stages that included (1) a national political commitment to reducing NCDs, (2) a situation analysis, (3) the mapping and recruiting of stakeholders, (4) a draft blueprint of national NCD plan, (5) multi-sectoral meetings, (6) monitoring and evaluation and (7) finalization and endorsement. Our novel MSC enhancement approach will work following these stages to produce policy-specific multi-sectoral action plans, but the difference here is that we will begin stage 1 with the commitment of the middle-level personnel and then slightly adjust the rest of the stages accordingly.

**Fig. 2:**
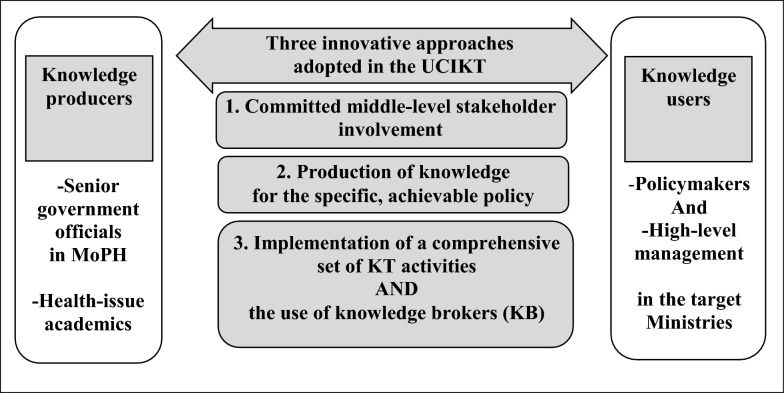
Conceptual framework of the User-oriented Comprehensive Integrated Knowledge Translation (UCIKT). KB, knowledge broker; KT, knowledge translation; UCIKT, User-oriented Comprehensive Integrated Knowledge Translation

Together with the concepts of knowledge translation and knowledge brokers, we formulated a novel MSC enhancement approach, namely the “User-oriented Comprehensive Integrated Knowledge Translation” (UCIKT) (See Fig. 2). It consists of three main concepts: (1) incorporating committed middle-level stakeholder involvement in MSC to overcome the constraint of a lack of commitment from high-level politicians (corresponding to stage 1 above), (2) producing knowledge for specific, achievable policies instead of for broad NCD prevention and control policies (corresponding to stages 2, 3, 4 and 6) and (3) applying the implementation of a comprehensive set of knowledge translation activities and the use of a knowledge broker instead of routine simple bureaucratic communication (corresponding to stages 5 and 7).

Performing MSC to advocate evidence-informed decision-making for non-health sectors to launch their policies to control NCDs needs two distinct connections: (1) a connection between knowledge producers and knowledge users and (2) a connection across relevant ministries. Therefore, we propose using intensely effective communication strategies in the UCIKT: applying Lavis et al.’s comprehensive set of knowledge-translation activities as an effective communication platform [6] and Dobbins’ concept of knowledge broker as an effective communication person [18, 19].

Lavis et al.’s framework includes push efforts, pull efforts, efforts to facilitate user pulls and exchange efforts [6]. The push efforts involve translating the produced knowledge to actionable messages. The pull efforts include the knowledge users “reaching out” to the research world to extract information regarding a particular decision they must make. Efforts to facilitate user pulls include a one-stop shopping information platform to locate optimally packaged systematic reviews, a rapid-response unit that provides written summaries, telephone or in-person consultations about the best research available, and the provision of it to the user in a timely manner. The exchange efforts relate to the development of a partnership between knowledge producers and knowledge users, allowing them to share their understanding about the necessary questions to ask, how the questions should be answered, and learning from one another.

Knowledge brokers (KBs) are individuals who connect knowledge producers (KPs) with knowledge users (KUs) because they have an understanding of the perspectives of both sides and are able to use appropriate language to communicate with both of them [18]. The roles for KBs include “knowledge managers” (e.g. create and disseminate knowledge), “linkage agents” (bridge knowledge producers and knowledge users) and “capacity builders” (e.g. train knowledge users) [19].

To implement the UCIKT in our research proposal, we have recruited university academics through Consultation Meeting 4. These were the academics who had experience working on NCD prevention and control with us previously and had shown a willingness to work on NCDs and a capacity to function as one of our KBs. Each KB (for each target-advocated policy) will form an MSC working group consisting of middle-level government officials from relevant ministries to work collaboratively to advocate for policy changes by producing knowledge supporting the specific target-advocated policies, operating a series of collaborative meetings (as exchange efforts) for information sharing and proposing policy recommendations finally. The non-health government officials will use the produced knowledge to inform the problem situations and policy recommendations to the high-level management in their respective ministries (as pull efforts).

The acronym UCIKT was arrived at as follows. Since the main approach to enhance MSC is knowledge translation (KT), a dynamic and interactive process that includes synthesis, dissemination, exchange and application of knowledge to improve health, provide effective health services and strengthen the healthcare system [17], by involving the key stakeholders from the beginning to the end, it becomes an integrated KT process [17]. The integrated KT process becomes comprehensive by following the knowledge translation framework proposed by Lavis et al. [6] and the use of knowledge brokers as proposed by Dobbins et al. [18]. Finally, the U in the acronym UCIKT stands for “user-oriented”. In this case, the KT was oriented for the senior non-health government officials as these individuals were allowed to select the specific health policies they wished to collaborate on.

### Results of the proposal development to implement and test the UCIKT

#### Results of brainstorming meetings

Table 1, the results of Meeting 2, provides a list of possible non-health-sector policy interventions for each of the risk factors and identifies multiple non-health ministries as potential collaborators (see columns). As the table demonstrates, there were multiple NCD-related policies requiring each non-health ministry to do work for the health sectors (see rows).

**Table 1:**
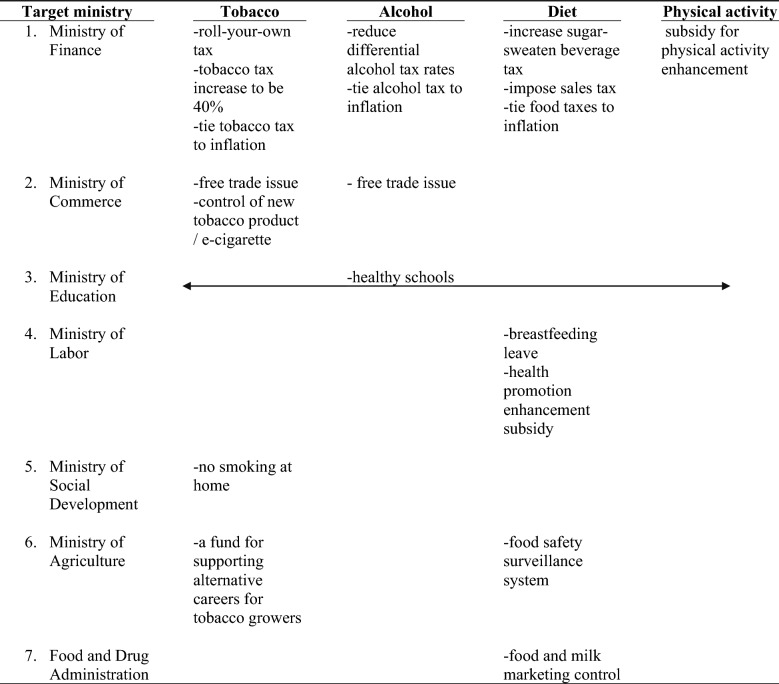
Priority health issues identified by the health sector

#### Final selection of specific risk-factor health policies for collaboration

Table 2, reflecting the results of Meeting 3, presents the ministry-specific policies chosen from the list of priority policies mentioned in Table 1 for collaboration by several of the senior non-health ministerial officials and the reasons for choosing them. These policies include (1) the integration of national surveillance systems regarding pesticide residuals on fresh vegetables and fruits (for MoA), (2) a taxation policy to control the use of chemicals in agriculture (for MoF) and (3) the integration of various risk-factor-specific extracurricular activities at the primary and secondary school levels (for MoE).

**Table 2:** Target NCDs-related ministerial policies that should be advocated for from the non-health ministry perspectives

Ministry	Target advocated policy	Rationale
1. Ministry of Agriculture and Cooperation	- The integration of the national surveillance systems regarding pesticide residuals on fresh vegetables and fruits	- Several small parts of the surveillance systems regarding pesticide residuals on vegetables and fruits in Thailand are lacking appropriate integration at the national level
2. Ministry of Finance	- Taxation policy to control the use of chemicals in agriculture	- Chemicals used in agriculture in Thailand are prevalent and can cause NCDs. Taxing chemicals strategically could be a good strategy to control their uses to prevent NCDs for Thai people
3. Ministry of Education	- The integration of various specific health-issue extracurricular activities at the primary and secondary school levels	- There are various issue-specific health extracurricular activities (alcohol, tobacco, diet, physical activity) in primary and secondary schools which lack appropriate integration

#### A summary of the research proposal to implement and test the UCIKT

On the basis of the logic model as proposed in the toolkit for developing a multi-sectoral action plan for non-communicable diseases by the WHO [20], our proposal’s objectives are to conduct research on the implementation stage to develop and evaluate contexts, inputs, processes and outcomes of the UCIKT. Our research questions cover (1) how the UCIKT works (examining the process), (2) what the outcomes and end-products of its impacts are, and (3) the identification of the contextual factors and inputs that influence the successes.

Planned research activities include a 3-month preparation phase, the UCIKT implementation phase for 15 months, a lessons-learned extraction phase for 2 months and data analysis and final report finalization for 4 months, totalling 24 months. The preparations include submitting an ethics approval application and preparing research teams at both the ministry level and the project level. The research team at the ministry level will focus on the implementation of the policy-specific UCIKT intervention while the research team at the project level will address the lessons learned from the general implementation of the UCIKT as well as any variations required due to policy-specific implementations.

The UCIKT implementation will be run by the knowledge brokers who will conduct policy-specific knowledge production collaboratively with all stakeholders, push efforts, efforts to facilitate user pulls and exchange efforts. The non-health government officials will implement pull efforts. The goal of UCIKT implementation is to enhance policy-relevant knowledge generation and evidence-based policy decision-making in the target non-health ministries.

Our research proposal’s expected outcomes are to produce three policy case studies that advocate for successfully implementing the UCIKT and providing a comprehensive summary of the knowledge regarding the UCIKT.

## Discussion

After conducting a literature review and brainstorming, we formulated the UCIKT approach: to enhance MSC by working collaboratively with the middle-level personnel from involved ministries, an approach which is less reliant on the strong commitment of high-level management. Following four consultation meetings with government officials in Thailand, we then developed a stakeholder-engaged research proposal to implement and test it.

Our approach does not require any commitment from high-level management to involve themselves with the MSC, as in many other recommendations [3–5, 15, 21]. This approach would not replace the conventional approach, but would enhance it. We hypothesize that countries using both MSC approaches (top-level and middle-level) would eventually increase their chances of achieving the SDGs.

Knowledge production is clearly important for many steps of the MSC, such as defining problems, finding solutions and evaluating results [22]. However, our proposed UCIKT addresses knowledge production for specific, achievable policies. After consulting with all stakeholders, we identified that NCD prevention and control policies can be divided into three distinct policy levels: (1) the overall NCD reduction at the national level (e.g. MSC action plans for NCD control), (2) the issue-specific NCD prevention and control measures (e.g. MPOWER measures for reducing the consumption of tobacco) that can be implemented by the non-health ministries and (3) specific NCD prevention and control measures that could generate mutual interest between health and non-health sectors (e.g. whatever specific policies that elicit interest from both sides). To address the lack of interest among various non-health sectors in preventing and controlling NCDs, [3, 7] we shifted our focus from levels 1 and 2 to level 3, as we believed this would capture the attention and commitment of the non-health government officials of the target ministry while the conventional MSC approach focussed normally on knowledge production supporting levels 1 and 2. For example, in developing our research proposal, instead of forcing the MoA colleagues to work on reducing pesticide residuals in fresh fruits and vegetables, we agreed to initially advocate for establishing the national surveillance system to monitor the pesticide residuals before moving on to more complicated tasks. Since the shared vision is one of the crucial successful factors [23], there should be commitments to collaboratively advocate for the agreed-upon target policies from both health and non-health sectors. This shared vision brought forth increased commitments from stakeholders in implementing a MSC [4].

Our proposal will examine whether knowledge brokering by KBs does in fact influence a successful UCIKT, since the function of KBs is a growing research field [19, 24]. If knowledge brokering is proven to be a positive factor, the Ministry of Public Health in a real-world situation could include a budget for sub-contracting, and use good recruitment practices and effective training to engage university academics to work on this role in collaborating with various target ministries. To make the knowledge brokering task a win–win situation for everyone involved, university academics could use this opportunity to run a research study on how knowledge brokering could affect MSC within a specific ministry for the prevention and control of NCDs.

### Limitations

There are four limitations in this study. First, the UCIKT approach’s success greatly depends on the abilities of the KBs since these people will be conducting the main activities of the UCIKT. Hence, ensuring a good selection process for these individuals, adequate training for them and that regular performance evaluations are conducted for them are all required. Second, the proposed UCIKT implementation research addresses advocating for policy formulations in targeted non-health ministries. However, it does not yet enhance MSC for policy implementation that needs a longer term of commitment and more resources from targeted non-health ministries. However, demonstrating a good working relationship between ministries and a successful policy formulation as a quick win for the initial MSC effort may improve the chance of achieving a longer-term MSC for policy implementation. Third, even though implementing an effective MSC is crucial for preventing and controlling NCDs, the successful MSC does not guarantee good population health. Finally, fourth, it is important to note that this summary article only provides an overview of the MSC-enhancement research proposal planning process and its outputs. The innovative approaches used in the UCIKT approach have not yet been proven for their effectiveness. It is imperative to secure funding and mobilize relevant stakeholders to support the proposed research. By doing so, we can test the effectiveness of the UCIKT approach and its potential for improving MSC. If the UCIKT approach proves to be a successful alternative, the Ministry of Public Health could incorporate it into the National NCD control MSC action plan (e.g. budgeting, recruiting and training for KBs). This is particularly crucial for achieving the global NCD targets within the specified timelines, both at the country and global levels.

In conclusion, this new approach for enhancing MSC, involving a change from securing commitments from middle management rather than from top management in the non-health sector, brings hope for achieving the SDGs for Thailand and perhaps for other countries as well.

## Data Availability

No datasets were generated or analysed during the current study.

## Abbreviations

CCS: Country Cooperation Strategy
CIHR: Canadian Institute of Health Research
DALY: Disability-adjusted life year
KB: Knowledge broker
KP: Knowledge producer
KU: Knowledge user
NCDs: Non-communicable diseases
MSC: Multi-sectoral collaboration
SDGs: Sustainable Development Goals
UNTG: Thematic Working Group on NCDs prevention and control
UCIKT: User-oriented Comprehensive Integrated Knowledge Translation
UNIATF: United Nations Inter-Agency Task Force on the Prevention and Control of NCDs
